# Self-Stable Precipitation Polymerization Molecular Entanglement Effect and Molecular Weight Simulations and Experiments

**DOI:** 10.3390/polym13142243

**Published:** 2021-07-08

**Authors:** Jiali Qu, Yi Gao, Wantai Yang

**Affiliations:** College of Materials Science and Engineering, Beijing University of Chemical Technology, Beijing 100029, China; gaoyi56555@mail.buct.edu.cn (Y.G.); yangwt@mail.buct.edu.cn (W.Y.)

**Keywords:** reactive molecular dynamics, self-stable precipitation, maleic anhydride, molecular weight

## Abstract

In this paper, we developed a reactive molecular dynamics (RMD) scheme to simulate the Self-Stable Precipitation (SP) polymerization of 1-pentene and cyclopentene (C5) with maleic anhydride (MAn) in an all-atom resolution. We studied the chain propagation mechanism by tracking the changes in molecular conformation and analyzing end-to-end distance and radius of gyration. The results show that the main reason of chain termination in the reaction process was due to intramolecular cyclic entanglement, which made the active center wrapped in the center of the globular chain. After conducting the experiment in the same condition with the simulation, we found that the distribution trend and peak value of the molecular-weight-distribution curve in the simulation were consistent with experimental results. The simulated number average molecular weight (Mn) and weight average molecular weight (Mw) were in good agreement with the experiment. Moreover, the simulated molecular polydispersity index (PDI) for cyclopentene reaction with maleic anhydride was accurate, differing by 0.04 from the experimental value. These show that this model is suitable for C5–maleic anhydride self-stable precipitation polymerization and is expected to be used as a molecular weight prediction tool for other maleic anhydride self-stable precipitation polymerization system.

## 1. Introduction

Our group proposed that α-olefin and maleic anhydride can undergo a self-stable precipitation polymerization reaction in a specific solvent environment [1,2,3]. A series of research works have been employed. Xing et al. studied the Self-Stable Precipitation polymerization process of Vinyl Acetate and Maleic Anhydride [4,5] and investigated the influence of reaction time, monomer concentration and ratio, and effect of the reaction medium on the morphology and size of the prepared polymer microspheres. Hao et al. used the SP system for the alternating copolymerization of styrene/maleic anhydride (St/MAn) and investigated the changes in the monomer conversion rate, microsphere size, and relative molecular weight of the copolymer with the reaction time during the polymerization process [6]. Liu et al. further extended the range of SP system monomers to a series of olefin monomers in the C4~C9 fraction of the petrochemical industry [7] and prepared polyolefin products containing acid anhydride functional groups, indicating that the SP polymerization process has universality. Furthermore, based on the analysis of polymerization thermodynamics and kinetics, the nucleation and growth mechanism of self-stable precipitation was proposed: the initial stage of the reaction mainly occurs in the solution phase; polymer chains will aggregate and gradually grow and nucleate out of solution. Subsequently, polymer chains are continuously deposited on the particles that were precipitated from the solution to eventually grow into balls. Wang et al. used a fluorescence self-imaging method based on aggregation-induced luminescence to monitor the process of particle generation and growth in SP polymerization solution [8]. This method confirmed the behavior of the abovementioned nucleation and growth process.

**Figure 1:**
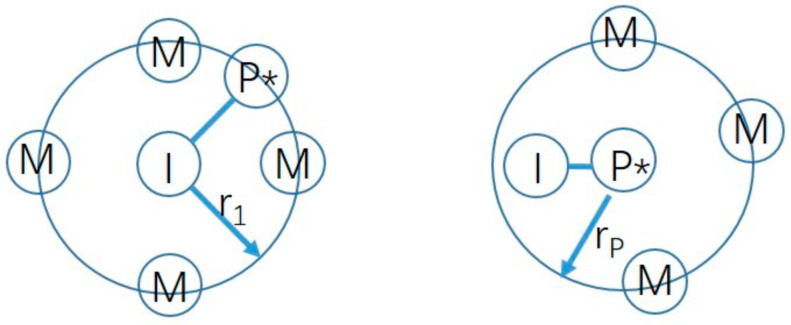
Schematic representation of the initiation step of the polymerization (left and center diagrams), as well as the chain propagation (right) (I is the initiator, P* is the active center of the growing chain, M is the unreacted monomer, r_l_ is the radius of initiation, and r_p_ is the radius of propagation) [11].

However, the influence of the entanglement/aggregation process of molecular chains in the solution phase in the early stage of the reaction has not been investigated, which is critical for revealing the mechanism in the molecular-level microscope. The main reason is that the size of molecules generated in the early stage of the reaction is very small, which is difficult to observe with current observation methods, and the chain growth process is extremely fast, which is difficult to research by sampling or in situ observation. However, MD simulation has great advantages in observing the small size and ultra-fast evolution process. It has been proved to be one of the most important tools to explore the mechanism in the field of self-assembly [9,10]. As to free radical polymerization, some researchers have recently used MD simulations to carry out related research [11,12,13], using Reactive Molecular Dynamics (RMD) methods to simulate the initiation and growth of polymers. The center diagram symbolizes the formation of a new I–P* bond (as shown in Figure 1). The two different reactive processes are characterized by capture radii r_I_ and r_P_. The reaction can take place whenever a free monomer bead M comes into the interaction sphere r_I_ of an initiator I*or r_P_ of a terminal reactive chain monomer P*. Torres et al. judged the termination of the reaction by introducing a gel point in the model [14]. Karim et al. studied the chain propagation process of styrene to monodisperse polystyrene in living polymerization [11]. Jiwon et al. further studied the molecular weight distribution of PMMA bulk polymerization and found that the simulated glass transition temperature and tensile modulus values were very close to the experimental results [15], which proved the validation of the method on simulating molecular weight and distribution. The above simulation studies all focus on bulk polymerization. There are few literature reports on precipitation polymerization simulation.

**Figure 2:**
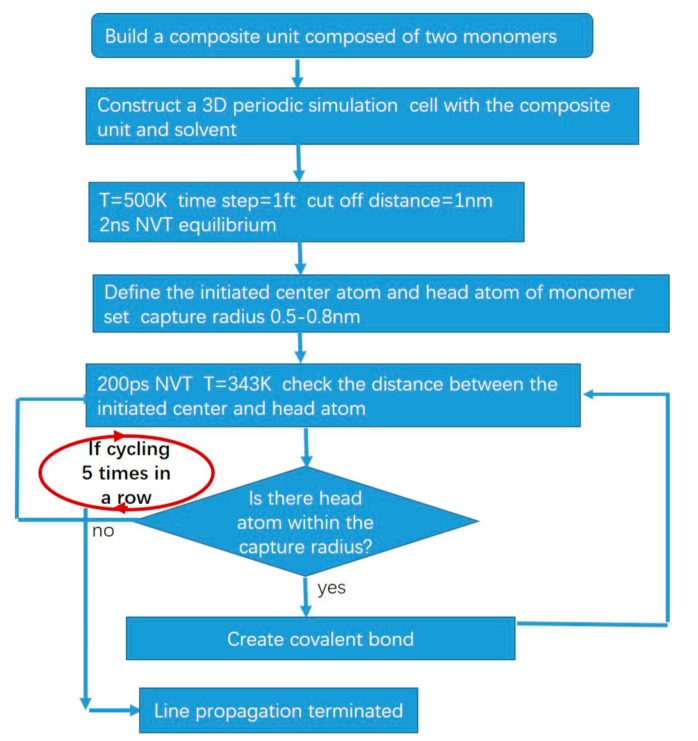
Scheme of molecular dynamics simulation algorithm for self-stable precipitation polymerization.

Through the prior experiments of our group, we found that the molecular weight of the copolymerization product of the C5–maleic anhydride reaction was much lower than that of common free radical polymerization. The degree of polymerization (DP) of the product was less than 100. In this study, the RMD simulation method was used to study two representative molecules 1-pentane and cyclopentene reaction with maleic anhydride. We investigated the process of chain growth and conformation evolution of the alternating copolymerization of pentene and cyclopentene with maleic anhydride, respectively, finding the mechanism for generating lower molecular weight product in this kind of SP polymerization. Correspondingly, we experiment with the same conditions as simulation in order to compare the simulated and experimental number average molecular weight (Mn) and weight average molecular weight (Mw) and the molecular weight distribution trend, which can validate the model we prepared. Since the number of free radicals in the early stage of the reaction is rare, the probability of free radicals connecting is small. To simplify the model and reduce the amount of calculation, only one active center was introduced in the model to study the evolution of molecular conformation during single-chain growth, the way of termination, and molecular weight distribution. Based on the algorithms proposed by Jiwon et al. [15], we developed the kinetic simulation schemes of SP reaction (shown in Figure 2), according to the alternating copolymerization theory [16] and the characteristics of the self-stabilizing polymerization reaction [5,7].

## 2. Simulation Method and Results

Since our simulation focuses on the early stage of the polymerization, the molecular weight is not high, DP < 100, and precipitation has not formed yet in solution. There is a different termination method from Jiwon’s in our algorithm. Jiwon’s termination condition is whether the conversion rate reaches a target value. Ours is whether the “no” loop cycles five times in a row; that is, if there are no head atoms within 1000 ps.

Construction of atomic model and RMD simulations were carried out with Materials Studio 7.0 software, Accelrys Company, (San Diego, CA, USA).

**Figure 3:**
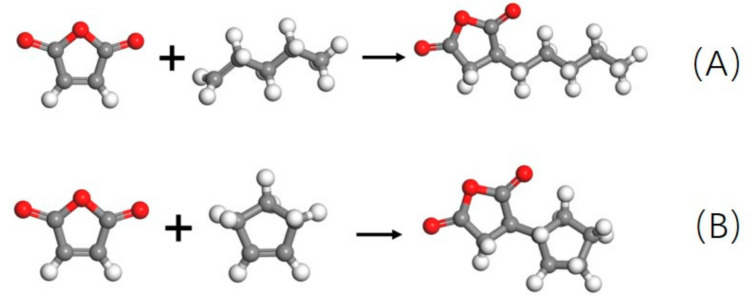
Maleic anhydride and 1-pentene composite monomer (**A**). Maleic anhydride and cyclopentene composite monomer (**B**).

**Figure 4:**
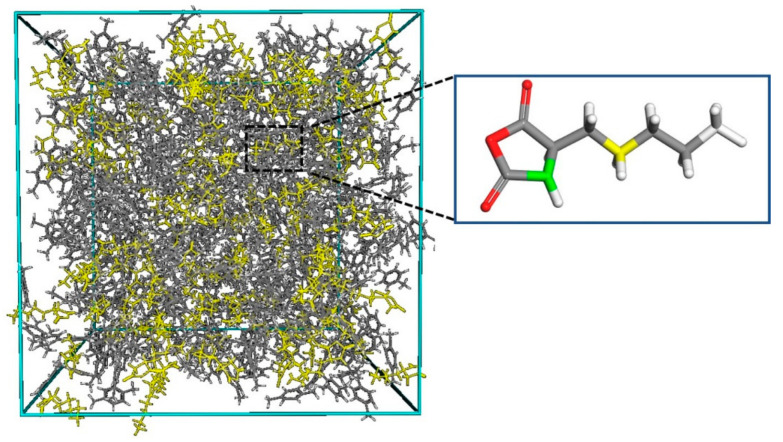
Cubic simulation cell (yellow parts represent composite monomers) and repeating unit structure (green atom is the initiated central atom; yellow atom is the head atom).

First, the double bonds in maleic anhydride and 1-pentene (cyclopentene) were changed to single bonds and linked to form a composite monomer, as shown in Figure 3. Then a three-dimensional periodic boundary cubic box composed of 200 composite monomers (the yellow part in Figure 4) and 1000 *p*-xylene molecules (a non-polar solvent) were constructed in the Amorphous Cell modules of Materials studio 7.0 software pack. The density was set to be 0.86 g/cm^3^ (measured in the experiment). The cell size was 64.7 Å × 64.7 Å × 64.7 Å. Two nanoseconds of NVT dynamic equilibrium was employed to the cell. The integration time step was 1 fs, the temperature T = 500 K, and the COMPASS force field was adopted, which was validated on alkane and phenyl liquid substances in the literature by Sun [17]. The temperature-control method adopted the Nose method to fully relax the system at a high temperature.

Then the carbon atom on the side of maleic anhydride in a composite monomer away from pentane was defined as the active center (the green atom in Figure 4). We performed 200 ps kinetic equilibrium at T = 343 K (the reaction temperature of the experiment) for chain relaxation [18,19] and then checked the distance between the active center and the head atom (yellow atom in Figure 4) of the unreacted monomer near the initiated monomer. The head atom with distance within the capture radius 0.5–0.8 nm was linked to the active center atom, realizing the chain growth process. The bond was formed with the closest one, if there were more than one such head atoms at each check step. If there was no matching head atom within this radius, we continued to perform 200 ps kinetic equilibrium and then checked again. If the process of no matching was repeated five times, the propagating line was terminated. That is, when no head atoms were found to enter the capture radius within 1000 ps, the reaction was considered to be terminated. The cell needs 1000 ps to reach an equilibrium state at the temperature 343 K, which was observed when the NVT equilibrium was employed before. Thus, we set this time length as a termination criterion. Thus, the polymer chain 1-pentene–maleic anhydride (1PM) and cyclopentene–maleic anhydride (CPM) were constructed.

**Figure 5:**
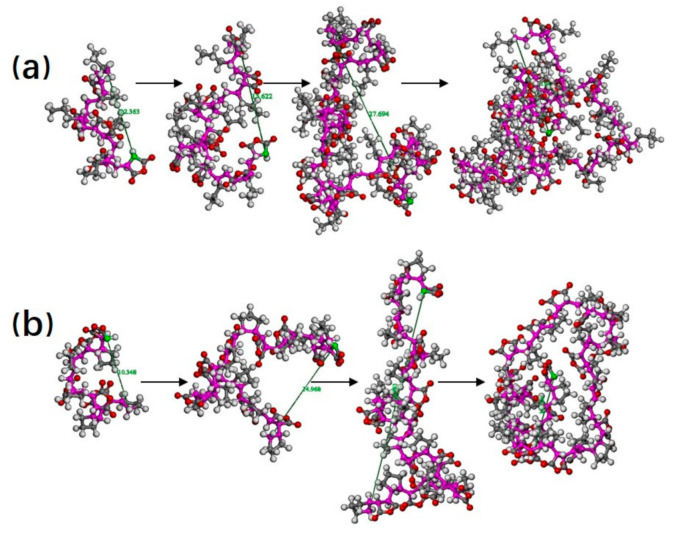
(**a**) Molecular conformation evolution of 1-pentene–maleic anhydride (1PM) during the growth process in the nonpolar solvent. The green atom is the active center, and polydispersity index (DP) is 5, 10, 20, and 30 from left to right, respectively; (**b**) the molecular conformation evolution of cyclopentene–maleic anhydride (CPM) during the growth process. DP is 5, 10, 15, and 20 from left to right, respectively.

Figure 5a is the snapshot of the molecular conformation captured at four different moments in the process of 1PM growth. From the figure, we can see that the conformation changes continuously during the chain growth process. The chain of 1PM keeps a line when the DP is 5 and 10. The molecular end-to-end distance and the radius of gyration (Rg) increase linearly from 12 to 18 Å when the DP is within the range of 5 to 15 (see Table 1). When the DP reaches 20, the line tends to circle. Simultaneously, the molecular end-to-end distance increases at a higher rate, from 18 to 27 Å. When the DP is up to 30, the extent of the circling enlarges. The line collapse to a globule, with the end-to-end distance plunging. The molecular chain turns to three-dimensional growth. From two-dimensional growth to three-dimensional growth, the chain undergoes collapsing and curling towards the center of the chain. We can call this process cyclization. The center is heavily wrapped at the end of chain growth and cannot continue to grow because of the lack of monomers nearby. Similarly, the conformation of CPM constantly changes during the growth process (Figure 5b). When the DP of CPM increases from 5 to 10, the end-to-end distance varies by the same changing rate as 1PM. As the DP increases from 10 to 15, the end-to-end distance varies from 15 to 30 Å, showing three-dimensional growth. It terminates when the DP reaches 20, with the end-to-end distance plunging by more than half. Moreover, the cyclization occurred in the polymer chain. From the growth process of these two chains, we find that, in the xylene non-polar solvent, the molecular chain has a strong tendency of cyclization that makes the chain to be a globule. When the chain grows to a certain extent, the active center is wrapped in the center of the globule, resulting in chain termination.

**Figure 6:**
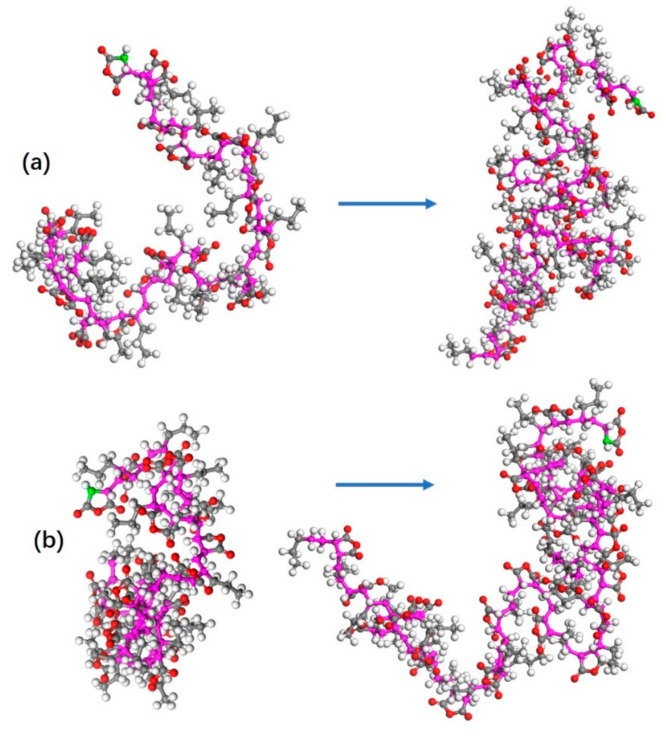
The molecular conformation evolution of 1-pentene–maleic anhydride (1PM) in the acetone as a solvent (**a**) and ethyl acetate as a solvent (**b**). The green atom is the active center, and degree of polymerization (DP) is 20 and 30 from left to right, respectively.

To investigate the influence of solvent on conformation, we employed the simulation mentioned above in two polar solvents, acetone and ethyl acetate, respectively. We found that the globular conformation does not occur to the 1CP chain in both of the solvents (see Figure 6). Moreover, the active centers were not wrapped. They can continue to grow until the monomers were rare. It is very likely that the non-polar solvent, *p*-xylene, leads to the formation of globular conformation at a low DP for this type of polymerization.

**Figure 7:**
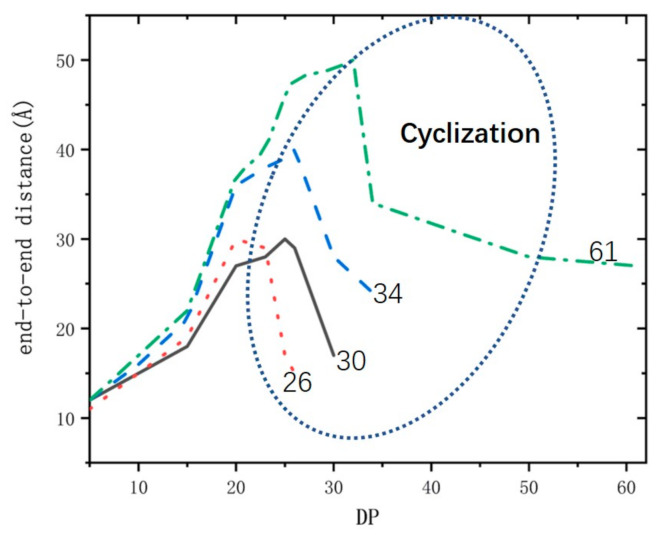
The end-to-end distance evolution of the 1-pentene–maleic anhydride (1PM) polymers with different polydispersity index (DP). Its values are showed at the end of the lines.

To study the distribution of molecular weight, more polymer chains with different molecular weights are needed. Due to the randomness of constructing cells in the AC module, we can obtain a new chain of 1PM when the initiation–growth–termination process was repeated. Repeat this process until 26 chains were simulated. Thus, we obtained a total of 26 polymer chains of 1PM as statistical samples. By the same method, we obtained 26 polymer chains of CPM. It was found that these chains all had the characteristics of intra-chain cyclization (see Figure 7) and active center being wrapped. When the active centers of the chains were wrapped earlier, the molecular weight of the polymers was relatively low, and when the active centers of chains were wrapped later, the molecular weight of the polymers was relatively high. It can be seen from Table 2 that the DP number of the copolymer 1PM ranges from 26 to 80, and the largest number of molecular chains (Ni) is 8 when the DP is 30. The DP of CPM ranges from 17 to 60, and the distribution of CPM ranges narrowly than that of 1PM. The highest Ni of CPM reaches 7 when the DP is 23. Based on the above analysis, the DP of CPM is smaller than that of 1PM. The copolymer chains of CPM have a greater tendency to cyclize than 1PM chain, so the chain termination of CPM occurs at a smaller degree of polymerization.

## 3. Comparison with Experimental Results

### 3.1. Experiment Procedure

Two grams of the monomer maleic anhydride and 0.5 g of the initiator AIBN were added into 12.5 mL of xylene solvent in a 100 mL flask and allowed to fully dissolve. Purged bubble nitrogen was used for about half an hour to remove the oxygen, and then 1.4 g of the monomer 1-pentene was added to the reaction flask. Then the obtained mixture was put into a 70b °C water bath for reaction, without magnetic stirring, the flask was sealed during the reaction. More experimental details and product structure confirmation were published [20]. In the experiment, the molar ratio of maleic anhydride to 1-pentene is 1:1, and the molar ratio of maleic anhydride to xylene is 1:5. The experimental temperature was also the same as the temperature during the simulation of chain growth. The reagents were all purchased from Alpha Reagent Company (Shanghai, China).

The liquid in the reaction flask remained clear as a solution state within ten minutes after the start of the reaction. Samples were taken out every one minute, and then the ten samples were taken for gel permeation chromatography (GPC) tests. GPC 1515-2414 detector produced by Waters company (Taunton, MA, USA) was used for the test, polystyrene (PS) as the standards, and tetrahydrofuran as the eluent. The samples were filtered through a 0.45 μm pore size syringe filter before sample injection. None of polymers were detected from the sample within the first 4 min, and there was polymer in the sample at the 5th minute. The polymer obtained at this time was used as the product of the early stage of the reaction for subsequent studies.

To verify the composition ratio of the copolymer, the acid–base titration method was used to determine the content of acid anhydride in the copolymer [21]. We took 0.018 g of the white copolymer powder after vacuum-drying into a 100 mL flask, added 20 mL of acetone, and heated to refluxed for about 10 min until the copolymer was fully dissolved. After cooling, we added an excess of KOH–ethanol standard solution (concentration of 0.0697 mol/L), and then heated to reflux for 5 min to ensure that the copolymer was fully dissolved. After cooling, phenolphthalein was used as an indicator, and the prepared HCl–isopropanol standard solution (solubility of 0.134 mol/L) was used as back titration; the amount of KOH–ethanol solution and HCl–isopropanol solution was 10.1 and 3.6 mL, respectively. We substituted them into Formula (1), and then GMAH = 0.6. Thus, the molar ratio of maleic anhydride and 1-pentene (cyclopentene) was 1:1, which proves that the obtained polymer was a regularly alternating copolymer.

(1) $$G_{\mathrm{MAH}}=9.806\left(C_{\mathrm{OH}}V_{\mathrm{OH}}-C_{H}V_{H}\right)/2m$$

where G_MAH_ is the mass fraction of MAn %; C_OH_ is the KOH–ethanol standard solution concentration, mol/L; V_OH_ is the amount of excess KOH–ethanol standard solution, mL; C_H_ is the concentration of HCl–isopropanol standard solution, mol/L; V_H_ is the amount of HCl-isopropanol standard solution in back titration, mL; and m___ is the mass of the copolymer sample, g. 

**Figure 8:**
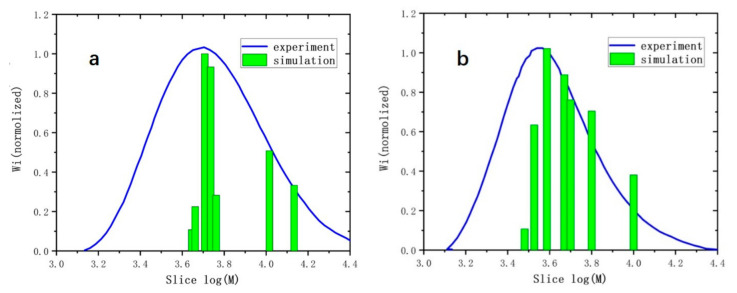
(**a**) Simulation and experimental molecular weight distribution of copolymer 1-pentene–maleic anhydride (1PM); (**b**) simulation and experimental molecular weight distribution of copolymer cyclopentene–maleic anhydride (CPM); the results were normalized to 1 for comparison.

The data obtained by GPC were relative values to the molecular weight of the standard PS, which can be corrected by the universal calibration method [22] to obtain the true molecular weight. We used the viscosity detection accessory of the waters GPC to determine the intrinsic viscosities (η) of each fraction of the copolymer and standard PS. Intrinsic viscosity data can be brought into Formula (2) to obtain the molecular weight, Mi, of each fraction. Then the simulated data and experimental data were used to plot the molecular weight curve (as shown in Figure 8), and then we used the Formula (3) to obtain the number average molecular weight (Mn), weight average molecular weight (Mw), and polydispersity index (PDI) (see Table 3).

(2) $${\left(\left[\eta \right]M\right)}_{i}={\left(\left[\eta \right]M\right)}_{\mathrm{standard}}$$

(3) $$Mw=\;\mathrm{WiMi}\;=\frac{\sum NiMi^{2}}{\sum NiMi}PDI=\frac{Mw}{Mn}$$

### 3.2. Comparison of Molecular Weight

From the distribution trend of the histogram (simulation) and the curve (experiment) in Figure 8, the peak positions of the simulation and the experiment are very close, indicating that the model can accurately simulate the molecular weight value of the largest number of fractions. Furthermore, the simulation and experimental results have a better consistency on higher molecular weight on the right side of the figure, indicating that the model can describe the behavior of the chain with larger molecular weight molecular generated by the reaction. The agreement in the distribution curve on the left is not ideal, because in order to improve the calculation efficiency, the model does not involve chain transfer termination [23] that takes place in the chains with low molecular weight in the growth process. Despite this, it can be seen from Table 3 that, for the copolymers 1PM and CPM, the difference between the simulated Mw and the experimental Mw is less than the molecular weight of one repeating unit, 170 g/mol, reaching the accurate value match. The reason is that the largest number of fraction and the fraction with larger molecular weight occupy a larger weight in the statistical average value Mw (see Formula (3)). The simulated PDI value is also consistent with the experimental value. For CPM copolymers with a smaller molecular weight, the simulated PDI value is 1.1, the experimental PDI value is 1.14, and the difference is only 0.04. From the above analysis, we can conclude that the algorithm used in this model is reasonable and can be used to present the chain growth and termination in the early stage of the self-stable precipitation polymerization of C5–maleic anhydride. Moreover, the termination method of most polymers is the single-radical termination with an active atom wrapped in the center of the molecular chain.

## 4. Conclusions

This paper proposed a new algorithm for simulating low-molecular-weight (degree of polymerization (DP) < 100) polymers in the early stage of Self-Stable Precipitation (SP) polymerization. Using this algorithm, the chain growth process of the reaction of 1-pentene and cyclopentene with maleic anhydride was simulated under the same conditions as the experiment. The process of the chain growth was stated by the conformation image and the changing of molecular end-to-end distance and the radius of gyration. It was found that the reason for the termination of the two chains 1-pentene–maleic anhydride (1PM) and cyclopentene–maleic anhydride (CPM) was the intramolecular cyclic entanglement; the active centers were wrapped in the center of the globular chains, which cannot make contact with other monomers; and, finally, the chains terminate at a low DP. This investigation revealed the mechanism of entanglement/aggregation of SP polymer chains on a microscopic scale. Furthermore, we simulated more molecular chains to obtain the molecular weight distribution. We compared the molecular-weight-distribution curve with that of the experiment. It was found that the simulated value in a limited sample can be consistent with most of the experimental curves. The experimental and simulated values of number average molecular weight (Mn), weight average molecular weight (Mw), and PDI were in good agreement between experiment and simulation. It was validated that the model is suitable for simulating the alternating copolymerization of C5–maleic anhydride, and the chain growth mechanism is reliable. This model may be suitable to a wider range of self-stable precipitation polymerization systems, and we will further explore it in the future research.

## Figures and Tables

**Table 1:** Molecular size parameters during polymerization process.

Chain	DP	End-to-End Distance (Å)	Rg (Å)
1PM1	5	12	5.0
1PM1	10	15	7.5
1PM1	15	18	10.1
1PM1	20	27	11.5
1PM1	25	30	11.6
1PM1	30(end)	17	10.2
CPM1	5	10	5.0
CPM1	10	15	7.5
CPM1	15	30	10.5
CPM1	20(end)	11	9.4

**Table 2:** Polymerization degree and corresponding number of molecules.

Polymer	DP	Ni
1PM	26	1
1PM	27	2
1PM	30	8
1PM	32	7
1PM	34	2
1PM	61	2
1PM	80	1
CPM	17	1
CPM	20	5
CPM	23	7
CPM	28	5
CPM	30	4
CPM	37	3
CPM	60	1

**Table 3:** Molecular weight and molecular weight distribution of simulation and experiment.

Polymer	Mn (g/mol)	Mw (g/mol)	PDI
1PM_simulation_	6001	7531	1.25
1PM_experiment_	5550	7660	1.38
CPM_simulation_	4581	5028	1.10
CPM_experiment_	4517	5150	1.14
